# Battle of the Bites: The Effect of Sewage Effluent Exposure on Mosquitofish Biocontrol of Mosquitoes in Residential Louisiana

**DOI:** 10.3390/toxics12040259

**Published:** 2024-03-30

**Authors:** Emily A. Kane, Shubham V. K. Yadav, Adeline Fogle, Nigel A. D’Souza, Nicholas DeLisi, Kevin A. Caillouët

**Affiliations:** 1 Department of Biology, University of Louisiana at Lafayette, Lafayette, LA 70503, USA; 2 Department of Environmental Studies & Sciences, Gonzaga University, Spokane, WA 99258, USA; dsouzan@gonzaga.edu; 3 Department of Biology, Gonzaga University, Spokane, WA 99258, USA; 4 St. Tammany Parish Mosquito Abatement, Slidell, LA 70460, USAkcaillouet@stpmad.org (K.A.C.)

**Keywords:** *Gambusia affinis*, body condition, toxicity, LD_50_, LT_50_, predation, masculinization, onsite wastewater treatment system

## Abstract

Mosquitofish, *Gambusia affinis*, are eponymous larval mosquito predators. Their ability to colonize and survive in habitats that are uninhabitable by other potential predators allows them to naturally manage larval mosquito populations in most ground pools they are present in. However, effluent from residential onsite wastewater treatment systems (OWTSs) appears to limit the presence of fish predators. This is especially problematic in Louisiana, where regulations allow the discharge of OWTS effluent into open drainage conveyances. To determine the effect of effluent on the capacity of mosquitofish for biocontrol in contaminated areas, we assessed the body condition metrics of populations from two effluent-exposed sites and two sites not exposed to effluent, determined the lethal effect of effluent-contaminated drainage water on fish, and measured the prey consumption rates in the presence of effluent. Female fish collected from effluent-impacted sites had a reduced somatic body condition and most females examined displayed masculinized anal fins resembling the male gonopodium structure. This trait was not seen in fish collected from the control sites and has not yet been documented in association with OWTSs or in the state of Louisiana. Fish from the control sites survived at effluent-contaminated water levels < 70%, and the prey clearance rates increased with dilution. Onsite wastewater treatment system effluent has significant effects on both the short- and long-term persistence of mosquitofish, their body composition, reproductive health, and larval mosquito consumption. These effects likely release mosquito larvae from suppression and may increase the threat of mosquito-transmitted pathogens in effluent-contaminated locations.

## 1. Introduction

The mosquito, *Culex quinquefasciatus,* is a considerable threat to humans and animals as the primary vector of lymphatic filariasis, West Nile virus, St. Louis encephalitis, and avian malaria [1]. Aquatic, air-breathing larvae of *Cx. quinquefasciatus* inhabit ephemeral, organically enriched, and often polluted aquatic habitats in tropical and semi-tropical environments [2,3]. These same environments are generally intolerable for most aquatic predators, resulting in unchecked mosquito populations and an increased risk to human health [2,3,4,5], including in low-income neighborhoods in southern Louisiana [6]. One such source of polluted habitats occurs in locations exposed to effluent from malfunctioning residential onsite wastewater treatment systems (OWTSs), including traditional septic systems and aerated treatment units (ATUs). The U.S. Environmental Protection Agency (EPA) estimates that 10% of OWTS units have failed at any given time, and that half of all installed units predate regulation [7]. This, combined with sewage overflow problems from combined sewer systems, where excess rainwater causes sewage to overflow [8], suggests that the impact of effluent on mosquito control could be a significant problem across the USA. For example, *Cx. quinquefasciatus* are prevalent inside of cracked or otherwise exposed septic systems across Puerto Rico [9], and areas susceptible to OWTS failure in Alabama have been positively associated with increased downstream *Escherichia coli* concentrations in the Choccolocco Creek watershed [10].

Due to heavy clay soils that do not allow wastewater to percolate, the state of Louisiana allows the direct discharge of grey and black water from residential OWTSs into drainage ditches. Among the parishes of Louisiana, St. Tammany Parish has the greatest number of OWTSs, which impact ~600 miles of roadside drainage ditches [11]. Traditional septic systems partially treat sewage by providing space for solids to settle and eventually be pumped out, as well as space for some anaerobic breakdown of wastewater products [12]. However, traditional septic systems that discharge effluent into open surface waters do not meet Louisiana regulations, and upon the inspection mandated at property transfer, must be replaced with an aerated treatment unit (ATU) [12]. Similar to a septic system, an ATU contains an air pump (requiring power) to promote the agitation and aerobic breakdown of the bacteria in wastewater. Both systems release partially treated sewage, and rely on additional “downstream” treatment for efficacy. The conventional treatment utilizes a trench or chamber system, where the partially treated water is diverted across pipes or gravel beds to continue treatment by soil or ground-dwelling microbes [12].

Raw effluent from malfunctioning OWTSs creates habitats that are inhospitable to fish predators and allow mosquito larvae to thrive [8], contributing to an increased risk of mosquito-transmitted pathogens. Mosquitofish (*Gambusia affinis*) are native to the Southern United States and are one of the many natural predators of mosquito larvae. Unlike other larger potential mosquito predators, they are unique in their high tolerance to the temperature, oxygen, and pH extremes indicative of poorer quality habitats [13]. Since these fish can reproduce quickly and readily inhabit areas depauperate of other natural control mechanisms, organized public health and mosquito management efforts around the world rely on the introduction of these fish to control immature mosquito populations [14]. However, effluent exposure likely pushes the physiological tolerance of fish and limits their contribution to biocontrol in polluted locations. The St. Tammany Parish Mosquito Abatement District has documented that high total dissolved solids (TDSs) are a primary predictor of fish absence [15], but the mechanism limiting fish presence is unknown. The presence of mosquitofish increases with distance from effluent point sources, suggesting that they can survive in moderately polluted sites, but it is unclear how much dilution is necessary to promote survival. Additionally, it is unknown whether their ability to capture prey or other traits are compromised, despite the ability of the fish to survive in moderately contaminated areas.

Our work indicates the limited efficacy of fish biocontrol and the introduction of fish into OWTS-contaminated, fishless sites. We hypothesize that OWTS effluent causes poor water quality that negatively affects mosquitofish survival and/or predation ability. As a first step toward understanding the physiological limits of fish in contaminated water, we use adult female mosquitofish from natural populations to (1) compare the body condition of fish from OWTS-effluent-exposed and control non-contaminated habitats; (2) measure the acute toxicity of effluent using lethal concentration (LC_50_) and lethal time (LT_50_) metrics; and (3) assess the live mosquito larvae capture success rates of fish tested in three effluent concentrations. Females were targeted because they are more numerous, larger, and have a greater influence on food webs [16]. 

## 2. Materials and Methods

### 2.1. Fish Collection

Female fish were collected from roadside drainage ditches in two neighborhoods in which houses rely exclusively on OWTS systems (effluent-exposed) and two neighborhoods in which houses rely on municipal sewer systems (control, non-exposed) throughout St. Tammany Parish. Water quality parameters for multiple sites within each neighborhood were first screened using a handheld YSI ProDSS multimeter (YSI inc., Yellow Springs, OH, USA) to identify sites to use for this study. Fish were then collected within a month of this screening (Table S1). Rain was minimal and no changes in the vegetation or water level were observed throughout the summer, suggesting that the sample site conditions were relatively stable throughout our study duration; the fish collected from two locations within each neighborhood were combined to maximize the representation of fish. Sites in Slidell, LA, USA (exposed; 30.29647, −89.84336), and Mandeville, LA, USA (control; 30.38133, −90.06611 and 30.38875, −90.05749), were sampled on 11 July 2023–12 July 2023 (pair 1), and sites in Covington, LA, USA (exposed; 30.4563, −90.06033), and Abita Springs, LA, USA (control; 30.47579, −90.03747 and 30.47669, −90.03401), were sampled on 19 July 2023–20 July 2023 (pair 2). The exposed sites were sampled on the first collection day and the control sites were sampled over both days to facilitate the collection of large numbers of fish (175 fish on Day 1, 75 fish on Day 2). Fish were collected using a mesh dipnet and transferred to an aerated 50 qt cooler for transportation back to the UL Lafayette Ecology Center in Carencro, LA, USA (30.30492, −92.0092). All procedures were approved by the University of Louisiana Institutional Care and Use Committee under protocol #2023-013-8717. Animal collection was performed under a scientific collection permit from the Louisiana Department of Fish and Wildlife (permit #SCP222).

### 2.2. Water Collection

For the toxicity and feeding experiments, effluent-contaminated water was collected locally from New Iberia, LA, USA, to minimize the daily transport time (30.03201, −91.92655). Although this parish tends to be more rural than St. Tammany, the ditches showed qualitatively similar patterns of depth, width, and mosquito presence/fish absence near active drainage pipes. Our source ditch (Figure S1A) was approximately 120 m long, with effluent release primarily on the east end through multiple inputs. Emergent vegetation was absent at the source, but became more prominent westward, matching the direction of water flow. Fish were not observed along the entire length of the ditch, which dried up at the road intersection. Our study considers this water to represent field conditions where effluent concentrations mirror those typically encountered throughout southern Louisiana. This ditch was chosen for the project source water due to its large size, which facilitated multiple water collection events.

### 2.3. Body Condition

On the first day of sampling at each of the 4 sites, the first 25 adult female fish caught from the holding cooler were euthanized immediately with an overdose of MS-222, followed by 95% ethanol as a secondary agent. Fish were then transferred to 70% ethanol for storage for up to 3 days. The total body weight (BW) and standard length (SL) of the fish were determined. Their ovaries were dissected and weighed to determine the gonad weight (GW). The somatic index (SI, also known as Fulton’s condition factor, K) was calculated first by using the total weight as ((BW/SL^3^) × 100) [17,18,19,20,21,22] and second by subtracting the gonad weight (eviscerated somatic index, SI_E_) to control for the influence of the gonad size as (((BW-GW)/SL^3^) × 100) [23]. These metrics are used as a proxy for nutrition and fish health, where, within a species or population, healthier fish show higher values [22]. The gonadosomatic index, or the percentage of body weight attributable to offspring development, was calculated as ((GW/BW) × 100) [23].

### 2.4. Effluent-Contaminated Water Toxicity

Toxicity assays using female fish from the control populations were set up based on the standardized Whole Effluent Toxicity (WET) methods developed by the US EPA [24]. In summary, fish from sites not exposed to effluent (control sites) were first acclimated to the environmental conditions set up in the lab. The acclimated fish were then exposed to a range of concentrations of OWTS-effluent-contaminated water and mortality was assessed at 8 h intervals over a 4-day exposure period. All surviving fish were euthanized at the end of the experiment. Further details are provided below.

Assays were conducted in a large outdoor greenhouse at the UL Lafayette Ecology Center in Carencro, LA, USA (Figure S1). The greenhouse was covered with a shade cloth and rain barrier, and was fitted with temperature-controlled ventilation fans. Additional protection from the sun and heat was provided by a popup canopy inside the greenhouse. Wire shelving was set up perpendicular to the air flow and between the flows from two large exhaust fans. Test containers were made using 34.6 × 21 × 12.4 cm transparent BPA-free and phthalate-free polypropylene storage bins with opaque lids (Sterilite Corporation, Townsend, MA, USA). Ventilation was provided by removing 4 panels along the long edges of the bins and above the water line, and gluing mosquito netting in their place. The bins were spaced approximately 7.5 cm apart and were filled with 3 L of dechlorinated tap water from a circulating holding bin. No filtration or temperature regulation was provided. A 2-inch PVC elbow connector was added to each bin to provide shelter and mitigate aggression. The water temperature and light availability were determined by the environmental conditions in the greenhouse and were continuously monitored during the testing periods using 2 Onset HOBO MX2202 data loggers (Onset Computer Corporation, Bourne, MA, USA) placed in two control (0% effluent) bins on opposite sides of the setup.

Upon return to the lab, 5 fish from the sites not exposed to effluent (control sites) were introduced into each test bin (total 105 fish) and allowed to acclimate to the test containers for 24–48 h. The remaining fish were placed into 38 L cycled aquariums with bubble filtration. The bins were inoculated with starter bacteria (API Quickstart, Mars, Inc., Chalfont, PA, USA) and ammonia conditioner (API AmmoLock, Mars, Inc., Chalfont, PA, USA) according to the label instructions on both acclimation days. Algal growth on the walls of the bins provided nutrition, and the fish were not supplemented with food. Fish in the holding tanks were fed flake (Fluval Bug Bites spirulina formula, Rolf C. Hagen Corp, Mansfield, MA, USA) and/or pellets (Ultra Fresh Royal Guppy Mignon Pellet, Taipei City, Taiwan) once daily to satiation. Ammonia was tested, and 90% water changes were performed daily for the bins and as needed in the holding tanks. Fish that died during the acclimation period were replaced with fish from the holding tanks.

Testing began at approximately 09:00 and lasted 91–96 h. Tests were conducted sequentially to minimize the exposure of personnel to extreme heat during daily maintenance; Mandeville fish were tested from 14 July 2023 to 18 July 2023 and Abita Springs fish were tested from 22 July 2023 to 26 July 2023. Water containing OWTS effluent contamination was collected from the source ditch at the location where the input pipes drained into the ditch. Freshly collected ditch water containing effluent was mixed with dechlorinated tap water at 7 concentrations: 0%, 15%, 30%, 45%, 60%, 75%, and 90%. Each treatment had 3 replicate bins, which were arranged on the shelves using a random number generator in Microsoft Excel (v. 16.77.1). We used a static-renewal exposure system in our experiments. Specifically, since the stability of effluent composition is unknown, 90% water changes were performed daily at 09:00 to maximize the potency of the effluent. Mortality was assessed every 8 h at 06:00, 14:00, and 22:00 each day (as well as during water changes at 09:00). Dead fish were removed, the SL and BW were recorded, and fish were preserved in 70% ethanol. During acclimation and Day 1 of the second test period (Abita Springs fish), temperatures reached extreme highs >40 °C and ice was added to the test bins at approximately 11:00 during acclimation and on the first test day to maintain consistent temperatures across all experiments (Figure S2) and to prevent fish from reaching lethal thermal maxima. The lethal maximum temperature for mosquitofish populations in Texas is 38–40 °C [25], and air temperatures were consistently at or above this maximum during the late summer. At the end of Day 4, all remaining fish were euthanized, the SL and BW were recorded, and the fish were preserved in 70% ethanol. 

Dose–response curves were generated based on the mortality observed during the experiment. For each population, the lethal concentration at the 50th percentile (LC_50_) of the freshly collected effluent-contaminated water was determined as the concentration at which survival was 50% of that observed in the controls. The lethal times (LT_50_) were determined as the time at which survival for each concentration exceeded 50% mortality of the starting population (2 fish or fewer remaining). The NOEC (No Observed Effect Concentration) and LOEC (Lowest Observed Effect Concentration) values were determined from the dose–response curves, representing the highest concentrations at which no significant mortality and the lowest concentration at which significant mortality were observed, respectively. The somatic index of fish was calculated for all fish for a comparison with the body condition analyses.

Biological oxygen demand (BOD) was monitored using opaque 300 mL glass bottles for select effluent concentrations 0%, 15%, 45%, and 75%. Bottles were placed on the wire shelving next to the respective bins with the same concentrations to ensure that they experienced the same environmental conditions as the test bins. Bottle temperatures and dissolved oxygen were measured at 0 and 24 h each test day. Water in the bottles was exchanged with fresh effluent daily, at the same time as test bin water changes. Oxygen drawdown over 24 h was determined by subtracting the final oxygen values from the initial values for each bottle on each day.

### 2.5. Prey Capture Behavior

The female fish remaining in the holding tanks following the toxicity assays were used to assess the rates of fish feeding on mosquito larvae in effluent-exposed water. Due to extreme heat conditions in the greenhouse creating an unsafe environment for both fish and personnel, the holding tanks were moved to a temperature-controlled room on the UL Lafayette main campus on 25 July 2023. To ensure consistency with the temperature used across all experiments in this study, aquarium heaters were used to maintain the tank temperature at 34 °C. Experiments with effluent-exposed water were conducted in an outdoor shaded area to ensure adequate ventilation. Mandeville fish (21 fish) were tested on 29 July 2023–30 July 2023 and Abita Springs fish (24 fish) were tested on 4 August 2023–5 August 2023 at approximately 10:30. Live 3–4 day old mosquito larvae were collected using an aquarium dipnet from the source water site in Iberia Parish and transferred to dechlorinated tap water. The mosquito age was approximated based on their morphology and size.

Fish were randomly selected from the holding tank and individually placed in a 20 × 7 × 14 cm custom-built acrylic arena containing one of three concentrations of freshly collected effluent-contaminated water: 0%, 45%, or 90%. Four replicates of each concentration (12 simultaneous tests) were arranged in an ordered, staggered pattern that was shifted each day to account for placement effects. After 1 h of acclimation, fifteen mosquito larvae were introduced into each arena and the number of larvae remaining was recorded every 15 min for 60 min. The prey clearance rate (%) was calculated as (#larvae eaten/#larvae introduced) × 100. The water temperature was 31–32 °C in the tanks during the 2 h of acclimation and testing. At the conclusion of testing, the fish were euthanized, the SL was measured, and the fish were discarded.

### 2.6. Statistical Analysis

For the water quality parameters, measurements were taken multiple times within each site (city) and sample location (house). The sample location values were averaged prior to performing statistical analyses, representing multiple sample sites within each neighborhood. The standard general linear models representing the *t*-test and analysis of variance (ANOVA) approaches were used to determine the differences in parameters between treatments, concentrations, and populations. Differences between treatments were performed across all 4 sites as well as for each sample pair. Tukey HSD tests were used to perform pairwise comparisons following the ANOVA results. Statistical analyses were performed in JMP Pro 15 (SAS Institute Inc., Cary, NC, USA) and Microsoft Excel (v. 16.77.1). To correct for multiple comparisons, a Benjamini–Hotchberg correction [26,27] was calculated for relevant tests. 

## 3. Results

### 3.1. Site Comparison

The control neighborhoods held considerably less water in the ditches and fish were collected primarily from larger permanent streams. The Abita Springs sites were fed by natural springs but the other locations were not. In the effluent-exposed sites, fish diversity was limited to live-bearing fish (*Gambusia affinis*, *Heterandria formosa*, and *Poecilia latipinna*). However, the control sites contained a greater diversity that included potential competitors (*Etheostoma* sp., *Aphredoderus sayanus*, and *Elassoma* sp.) and aquatic predators (*Lepomis* sp., *Esox americanus*, and *Siren intermedia*). Although the exposed sites showed decreased oxidation–reduction potential (ORP) and increased conductivity, salinity, and total dissolved solids (TDS) compared to the control sites, the differences were not significant once they were controlled for multiple comparisons (r^2^ < 0.236, *p* > 0.02). However, this pattern was driven by differences in these parameters within the Mandeville/Slidell pair, which did retain significance following correction; no differences were observed between the Covington/Abita Springs pair (Table 1). The Slidell site appeared to be in the worst condition, with the lowest ORP and highest conductivity, salinity, and TDS. 

### 3.2. Body Condition

**Figure 1:**
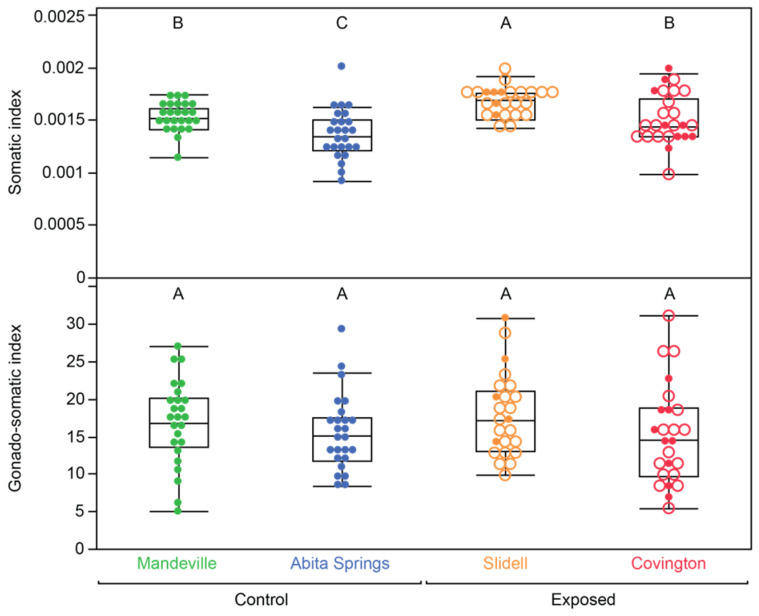
Body condition of female mosquitofish captured in four natural populations. Colors represent the population of origin. The somatic index was calculated using the total body weight, including the gonad weight. Open circles denote masculinized fish. Letters indicate statistical similarity, determined using Tukey HSD post hoc comparisons.

The control fish were up to 18% longer and up to 50% heavier than the effluent-exposed fish, but the exposed fish showed an approximately 9% increase in the somatic index compared to the control fish (Figure 1; Table S2). All metrics except GSI showed significant differences between treatments, which were retained after correction for multiple comparisons (pooled tests, r^2^ < 0.15, *p* < 0.02). However, this trend was driven by the Mandeville/Slidell pair; no traits were significantly different in the Abita Springs/Covington pair (Table 2). Both somatic index metrics showed similar outcomes, but statistical significance was achieved more consistently using the standard index compared to using the eviscerated weight index. For this reason and because only the standard metric was obtained in subsequent tests, we focus on the standard metric for further analyses and discussion.

When the size metrics from subsequent tests were considered, it became apparent that, although we attempted to randomly sub-sample fish for body condition analysis, these fish were not representative. Specifically, in both control populations where additional fish were measured, the standard length was up to 11% smaller (Mandeville: F_2,150_ = 0.52, *p* = 0.5986; Abita Springs: F_2,148_ = 4.88, *p* = 0.0089) and the somatic index was up to 24% lower compared to fish from other analyses (Mandeville: t_127_ = 5.74, *p* < 0.0001; Abita Springs: t_125_ = 7.99, *p* < 0.0001). The inclusion of additional individuals in the control populations resulted in these fish having a greater somatic index than the respective exposed populations (Mandeville/Slidell: t_151_ = −2.35, *p* = 0.0198; Abita Springs/Covington: t_150_ = −3.24, *p* = 0.0015), in contrast to the result from the body condition analyses alone. 

**Figure 2:**
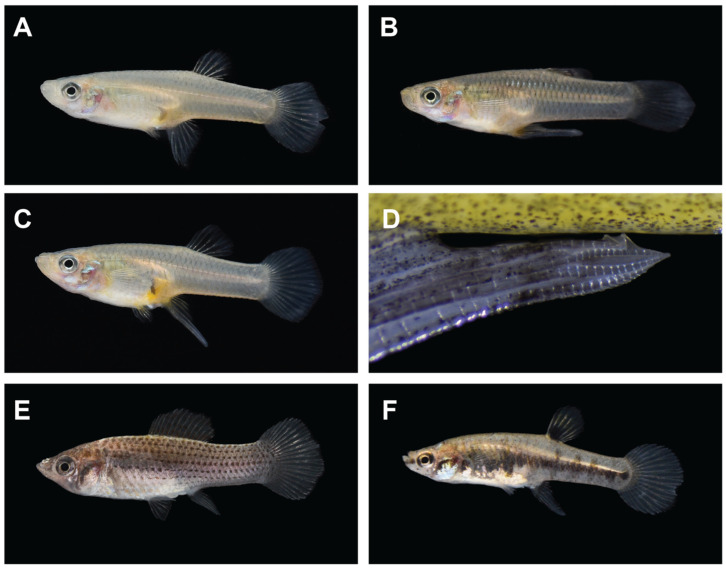
Masculinized females of three sympatric live-bearing fish species were found in effluent contaminated sites. (**A**) Typical female mosquitofish (*Gambusia affinis*) display a soft triangular anal fin and black breeding spot, whereas (**B**) typical males display a more rigid, fused, elongated anal fin that is modified into a gonopodium structure. (**C**) Masculinized female mosquitofish show a fused, elongated anal fin similar to males, while retaining a black breeding spot typical of females. (**D**) Fully masculinized anal fins develop terminal barbs typical of male gonopodia. Masculinization was also opportunistically observed in Iberia Parish mosquitofish as well as females of the other live-bearing species present in contaminated sites: (**E**) sailfin molly (*Poecilia latipinna*) and (**F**) least killifish (*Heterandria formosa*).

In addition to the traits quantified, 64–79% of females in the effluent-exposed populations displayed an anal fin with a morphological modification resembling the male gonopodium structure (Figure 2; 19 of 24 Slidell fish and 16 of 25 Covington fish). Specifically, the first few anal fin rays were elongated and fused, giving the appearance of a larger, more pointed anal fin. Masculinized females were not observed at the control sites. Within the exposed sites, masculinized females were larger than non-masculinized females in all length and weight measures, but did not differ in their body condition indices (Table 3). More extremely, nearly full anal fin elongation with a gonopodial spine (Figure 2D) was commonly observed in the Slidell fish, whereas the Covington fish displayed only slightly elongated anal fin rays. Sacrificed masculinized fish from both locations contained ovaries with embryos, but the developmental stages were not quantified. A sub-sample of live masculinized females from both exposed populations was brought back to the lab and observed in clean water for several months. Masculinized females did not revert to the non-masculinized phenotype within this time and offspring were not observed (but the setup was not conducive to this). Upon further qualitative surveying efforts, masculinized females were consistently observed at contaminated sites in both the St. Tammany and Iberia Parishes, as well as in both additional sympatric live-bearing species (*Poecilia latipinna* and *Heterandria formosa*) at the St. Tammany Parish contaminated sites (Figure 2).

### 3.3. Effluent-Contaminated Water Toxicity 

**Figure 3:**
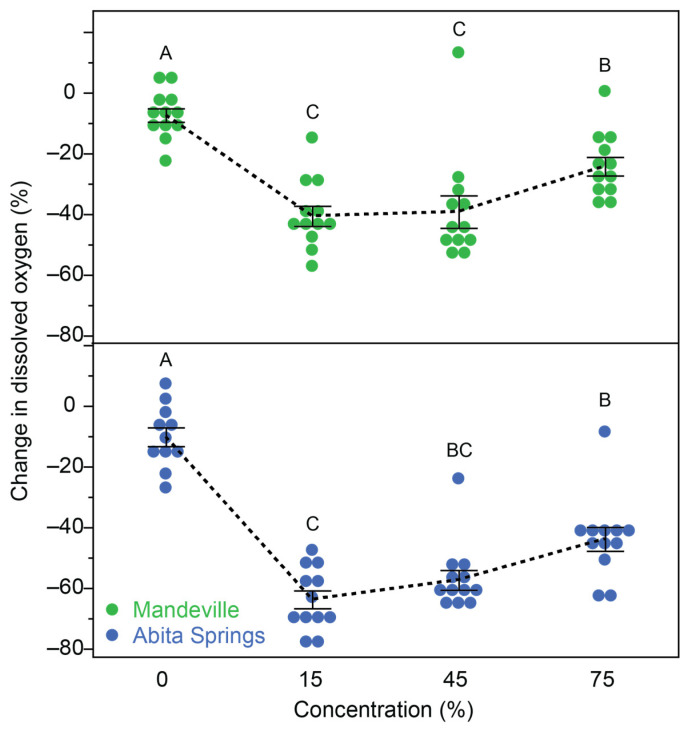
Biological oxygen demand measured from effluent-contaminated water estimated over 24 h. The change in dissolved oxygen for each concentration is represented as the mean and standard error of each measurement taken across three replicate bottles and four replicate days for each population. Colors represent the population of origin. Mean values are connected with a black dashed line to highlight changes. Letters (A, B, C) indicate statistical similarity, determined using Tukey HSD post hoc comparisons. In both populations, biological oxygen demand was greatest (most negative) at intermediate concentrations.

Both previously non-exposed, control populations of fish experienced similar levels of daily mortality during the effluent exposure trials. When deaths were observed, these occurred most often at the 14:00 checkpoint following a water change and tapered off over 24 h. Similarly, water appeared the darkest and most opaque immediately after the water change and cleared substantially in 24 h (Figure S1), suggesting the settling of particles or other changes in the water condition. The water temperature was similar across representative test bins during and across the testing sessions: the temperature ranged from 24.66 to 39.81 °C during the first test (Mandeville) and from 23.89 to 41.18 °C during the second test (Abita Springs; Figure S2). The biological oxygen demand was greater in the effluent water compared to the control and was greatest at intermediate 15% and 45% concentrations (Figure 3; Mandeville: ANOVA: F_3,44_ = 18.88, *p* < 0.0001; Abita Springs: ANOVA: F_3,43_ = 39.62, *p* < 0.0001), suggesting a reduction in microbial activity both in clean water and at the highest concentrations of effluent. This result was consistent across all 8 days of measurements (Figure S3). 

**Figure 4:**
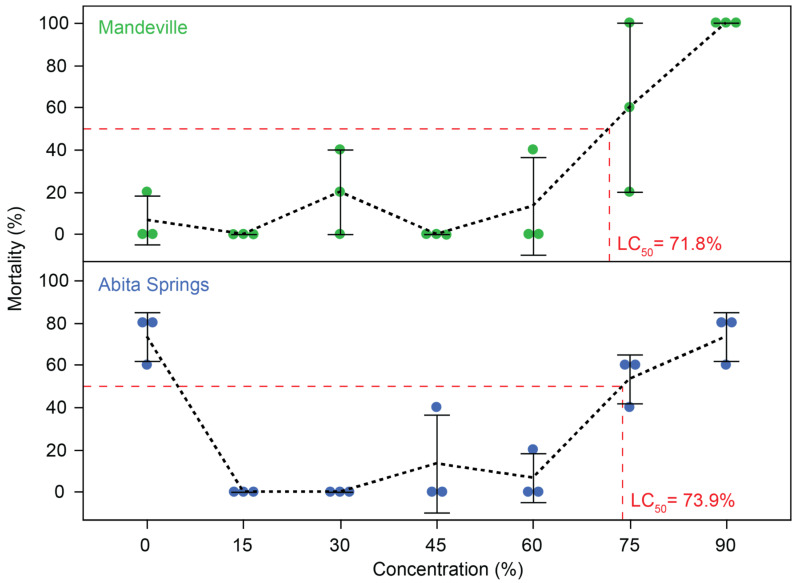
The effect of effluent-contaminated water exposure over four days on the survival of female mosquitofish (*Gambusia affinis*). Survival is represented as the mean and standard error of percent mortality for each of the three replicate tanks per concentration. Colors represent the population of origin. Mean values are connected with a black dashed line to highlight changes. The lethal concentration where fish experience 50% or higher mortality (LC_50_, red dashed lines) is similar between test populations.

While both test populations exhibited similar LC_50_ values of around 73% effluent concentration (Figure 4), an unexpected phenomenon was observed in the fish collected from the Abita Springs site. In the control group (0% effluent), fish exhibited high mortality (approximately 73 ± 11.5%) due to unusually high interspecific aggression (Figure 4). This behavior subsided in the presence of effluent (a stressor) and in the holding tanks (likely an individual dilution effect), and was not observed in the fish collected from the Mandeville site. The control water used in the experiment was also utilized in the holding tanks, where no mortality occurred. Taken together, these observations strongly suggest that the high mortality observed in the Abita Springs control group was an artifact unrelated to effluent exposure. Consequently, the 0% effluent concentration was excluded from the toxicity analysis for this population to ensure the accurate representation of effluent effects. Even with this non-traditional step, the results were consistent between populations, suggesting that the effect on our results was minimal. Mortality due to effluent was evident at or above concentrations of 30%. Within populations, fish that died during the test had body lengths, weights, and somatic indices that were similar to fish that survived the trial (Mandeville: t_100_ < 0.45; *p* > 0.65; Abita Springs: t_102_ > −1.54; *p* > 0.12). Although both populations had similar lengths and weights, Mandeville fish had a somatic index that was 7.6% greater than that of the Abita Springs fish (Table S3). 

Our results suggest NOEC values of 75% and 60% effluent-contaminated water for the two populations tested, with LOEC values of 90% and 75%. For the Mandeville fish, statistically significant mortality only occurred at the highest (90%) concentration tested (t_4_ = −14.000, *p* < 0.001), despite some mortality appearing at a 30% effluent concentration (t_4_ = −1.000, *p* = 0.374). For the Abita Springs fish, the high mortality observed in the control tanks necessitated setting the NOEC and LOEC relative to the next lowest exposure at which no mortality was observed (15% effluent). While some mortality occurred at 45% and 60% effluent-contaminated water concentrations (t_4_ = −1.000, *p* = 0.374), statistically significant mortality was observed only at elevated concentrations of 75% and 90% (75%: t_4_ = −8.000, *p* = 0.001; 90%: t_4_ = −11.000, *p* < 0.001). This indicates a relatively high tolerance to acute (4-day) effluent exposure in both populations of fish.

**Figure 5:**
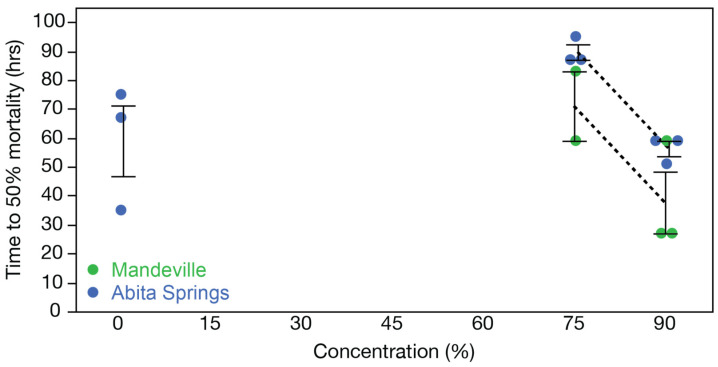
The effect of effluent-contaminated water exposure over four days on the duration of survival of female mosquitofish (*Gambusia affinis*). The mean and standard error of time to 50% mortality (LT_50_) are shown for each of the three replicate tanks per concentration. Colors represent the population of origin. Mean values over sequential concentration changes are connected with a black dashed line to highlight changes; concentrations that did not experience at least 50% mortality are not shown. The LT_50_ is similar between test populations and decreases with increasing concentration.

The LT_50_ of exposure in each concentration was similar between populations (Figure 5). Survival was high between 0 and 60% effluent-contaminated water, with 50% mortality only encountered at concentrations of 75% or above. As expected, the highest effluent-contaminated water concentration (90%) triggered the fastest population declines, with Mandeville fish enduring for 37.7 ± 18.5 h, and Abita Springs fish persisting for 56.3 ± 4.6 h. However, no significant differences in LT_50_ were observed in fish from the two populations following exposure to elevated sewage effluent concentrations (75%: t_3_ = −1.948, *p* = 0.147; 90%: t_4_ = −1.698, *p* = 0.165). Within each population, the LT_50_ in the Abita Springs fish was significantly lower for fish exposed to a 90% effluent-contaminated water concentration compared to those exposed to a 75% effluent concentration (t_4_ = 8.839, *p* < 0.001). In contrast, no significant differences in LT_50_ values were seen in the Mandeville fish exposed to either 75% or 90% effluent-contaminated water concentrations (t_3_ = 2.030, *p* = 0.135). Taken together, these results suggest that the effluent exposure tests were repeatable and that the strongest effluent-contaminated water concentrations are lethal in only 1–2 days.

### 3.4. Prey Capture Behavior

**Figure 6:**
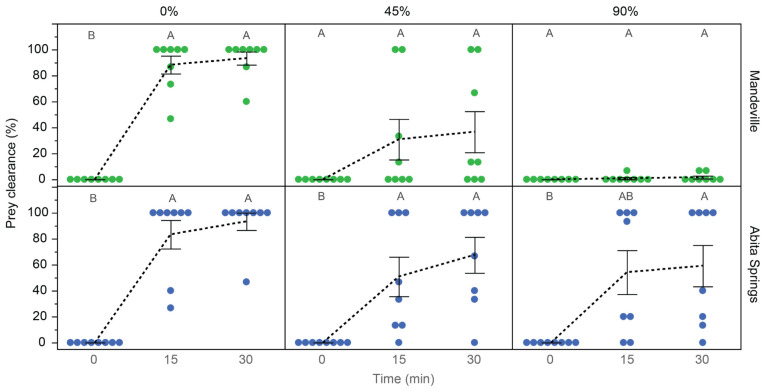
Female mosquitofish (*Gambusia affinis*) prey clearance rates in the presence of three concentrations of effluent-contaminated water. Prey clearance rates represent the percentage of prey removed at each time point. Only the first 30 min are shown because this is where the greatest effect was observed. The mean and standard error for each time point are shown for four replicate tanks across 2 replicate days. Colors represent the population of origin and letters indicate statistical similarity within each concentration and population. Mean values are connected with a black dashed line to highlight changes. In both populations, prey clearance is greatest in the first 15 min and is most significant at a 0% effluent-contaminated water concentration.

Feeding rates were highest at a 0% concentration and decreased with an increasing effluent-contaminated water concentration (Table 4, Figure 6). Fish tended to clear most prey within the first 15 min (Figure 6). At 30 min, Mandeville fish showed significant differences between the concentration treatments (Mandeville: F_2,21_ = 23.13, *p* < 0.0001; Abita Springs: F_2,21_ = 1.95, *p* = 0.1673), such that prey were almost completely cleared in 0% unexposed water, approximately 40% cleared in the 45% exposure treatment, and almost untouched in the 90% exposure treatment. Additionally, fish in the highest and lowest concentrations usually responded with little variation among individuals. In contrast, fish in the 45% concentration treatments showed a wide range of responses that may be due to individual effects or preferences. These patterns were most clear in Mandeville fish due to a substantial residential influx of freshwater into the source ditch during the tests with Abita Springs fish that led to the unexpected dilution of the effluent source water. This artifact is correlated to higher feeding rates in all Abita Springs fish, especially at a 90% effluent-contaminated water concentration. This influx was steady for at least 3 days, during which time we located the source and noted continued outflow. Based on water turbidity, we estimated that the day 1 tests were slightly diluted and that the day 2 tests were significantly diluted compared to the prior Mandeville population tests (Table S4).

The fish standard length was similar across days within populations (Mandeville: t_22_ = 1.05, *p* = 0.3067; Abita Springs: t_22_ = −0.61, *p* = 0.5471). However, despite randomly populating the observation chambers, fish exposed to 90% effluent tended to be smaller than fish in the other chambers (Mandeville: F_2,21_ = 4.15, *p* = 0.0303; Abita Springs: F_2,21_ = 2.99, *p* = 0.0720), but this outcome was not retained after correction for multiple comparisons. The standard length of the fish was similar between populations (t_46_ = 1.26, *p* = 0.2138). 

## 4. Discussion

Our study examined the effect of residential onsite wastewater treatment system (OWTS) effluent, such as is commonly found throughout southern Louisiana [11], on the effectiveness of mosquitofish (*Gambusia affinis*) predation on mosquito larvae. We found that (1) effluent-exposed sites vary in their water quality parameters, but generally have more dissolved particles in the water (as indicated by TDS and conductivity); (2) the highest concentrations of effluent-contaminated water are acutely toxic, leading to mortality in as little as 24 h; and (3) although fish can survive in diluted effluent concentrations, they exhibit additional non-lethal detrimental effects that include reduced prey capture behavior and long-term changes in their reproductive morphology suggestive of endocrine disruption. Both fish populations exhibited striking similarities in having a relatively high short-term survival in sewage effluent. Further, the presence of mosquitofish in more dilute areas (such as with increasing distance from effluent pipes) likely provide some benefit, especially compared to a complete absence of fish. However, fish likely have more stable populations and a more robust capacity for biocontrol in non-polluted locations.

The contamination of local water bodies from OWTS can be found throughout the United States and can be a significant problem for mosquito management. For example, the St. Lucie Estuary in Florida has received significant public attention due to the downstream effect of OWTSs, resulting in elevated nitrogen, phosphorous, and fecal loading contributing to blooms of the toxic cyanobacterium *Microcystis aeruginosa* [28]. Re-occurring brown tide *Aureoumbra lagunensis* blooms in the Baffin Bay estuary in Texas, a result of excess dissolved organic nitrogen, were also recently linked to sewage discharge [29]. But these water bodies are large and are potentially continually flushed, aiding recovery. In urban residential areas, the impact of OWTS contamination can be exacerbated when disease vectors such as *Cx. quinquefasciatus* take advantage of fishless, polluted, standing water [9]. Our results suggest that the greatest effectiveness of mosquitofish for biocontrol in effluent-exposed habitats is limited to moderately polluted locations. Therefore, the effective biocontrol of larval mosquito populations by mosquitofish will require enhanced wastewater management practices in regions affected by effluent exposure. However, our work utilized fish from control populations and it is possible that exposed fish could be locally adapted to effluent, such that their survival and tolerance are greater than what we observed here. Localized population-level genetic changes as a result of environmental stressors are a common response of both mosquitofish specifically and live-bearing fish more generally and have been observed, for example, in response to salinity in coastal Louisiana and to toxic substances in H_2_S springs [30,31,32].

### 4.1. Effluent-Contaminated Water Acute Toxicity

Notably, mortality only manifested above ditch-sampled effluent concentrations of 30%, with LC_50_, NOEC, and LOEC values all above 60% concentration. Additionally, the LT_50_ suggests that fish from both populations can survive for 1–2 days in effluent-contaminated water concentrations at or above 75%. These two populations indicate the remarkable tolerance of the fish to acute effluent exposures. This tolerance is similar to the lethal concentration (LC_50_) observed for much larger carp fingerlings (*Labeo rohita*) tested in untreated municipal sewage wastewater [33] and snakehead (*Channa punctatus*) tested in paper mill effluent [34]. However, after 4 days, most fish in our experiments exposed to 90% concentration experienced mortality, suggesting that long-term (lifetime) survival at these concentrations is not possible. Additionally, the toxicity of effluent is expected to be variable both spatially and temporally. For example, toxicity is greater for *L. rohita* (LC_50_ = 44%) and catfish (*Heteropneustes fossils*; LC_50_ = 6%) in sewage wastewater samples from an alternative location [35,36]. Similarly, we expect that toxicity is highly variable among OWTS effluent sites and fish populations in Louisiana. For example, at the end of our feeding study, we attempted to perform additional trials using a different effluent source ditch in Iberia Parish, only a few meters from the primary ditch. Fish mortality was surprisingly observed within minutes instead of hours at all dilution levels of effluent, suggesting increased toxicity compared to the water used in this study. It was not possible to characterize the inherent toxicity of OWTS effluent or the precise concentration of OWTS effluent in the water collected from the polluted ditches across the sites in this study, but is it likely that the contamination level is dependent on several factors that vary in both space and time.

We suspect that the mechanism of acute toxicity may be related to the toxicity of one or more unknown constituent particles or dissolved substances in the water. Our finding that dissolved particles are elevated in exposed sites is consistent with the observation that the total dissolved solids is the best predictor of fish absence in wastewater sites [15]. These factors may include high ammonia, asphyxiation from particulates or increased mucous production, or a toxic chemical constituent such as soap, pesticides, or pharmaceuticals [34,37,38]. Although the ammonia levels were extremely high in the undiluted effluent (measured qualitatively with an aquarium test kit), a symptom of ammonia toxicity that we have observed previously in mosquitofish in the lab is the reddening of the caudal peduncle, but we did not see this in the test bins. Additionally, we did not note surface gulping behaviors or excess external mucous production in the fish exposed to effluent, but we did not examine the gill tissue where mucous may have built up. One mechanism suggested to be associated with the acute toxicity of wastewater is interference with or damage to gas exchange and oxidative metabolic processes [34,36,37]. Fish exposed to the highest concentrations of effluent in our experiment often showed reduced movement throughout the tank, suggesting that a similar mechanism of metabolic impairment may result from our local sources of effluent.

We hypothesize that the absence of fish in high-concentration effluent regions is due to the behavioral avoidance of these areas. Fish can survive for several hours at toxic levels, providing time to relocate to a more suitable habitat. Additionally, the control fish experienced an immediate aversion to prey in the presence of effluent, suggesting that fish can detect effluent and are aversive to it. However, we are unable to determine whether toxicity results in immediate physiological changes or whether other effects such as reduced visibility trigger this response. 

### 4.2. Effluent-Contaminated Water Chronic Toxicity

Although fish demonstrate high survival in moderately contaminated effluent-exposed water, the long-term costs of living in this environment include reduced body conditions, the masculinization of adult females, and reduced feeding rates. Combined, these effects suggest that the long-term persistence of mosquitofish in these habitats may be less stable [39], and exposed populations may depend on increased reproductive effort or migration from non-exposed sites. However, behavioral responses such as learning or minimizing exposure to effluent, as well as physiological plasticity or adaptation in response to effluent exposure, may mitigate this constraint. Fish from the Slidell site showed the greatest degree of masculinization, suggesting that this site is either contaminated to a worse degree or contaminated more regularly. Therefore, the degree of masculinization in mosquitofish may serve as an indicator of pollution magnitude [40,41]. 

Mosquitofish from the effluent-exposed sites in St. Tammany Parish had a somatic body condition that was reduced by up to 11.5% compared to the control fish, but this was not due to the gonad weight and gestation. Therefore, these fish may prioritize reproductive effort with a cost to somatic growth, similar to the response observed in mosquitofish exposed to stressors such as increased salinity or simulated predation [23,42]. Our values, including the effect of reduced SI in polluted water as well as the magnitude of this effect, are similar to those found for mosquitofish and other fish more broadly [21,43,44,45]. Body condition metrics such as the SI and GSI are indicators of fish health, and larger values represent access to better nutrition [22] and a better ability to escape predators [46]. The moderately exposed areas that fish were collected from for this study sustained a complex ecosystem, suggesting that ample prey may be available in these habitats, likely due to eutrophication. Therefore, the reduction in SI observed may be more indicative of physiological or developmental constraints. For example, the SI was reduced compared to the control fish when female mosquitofish treated with the synthetic steroid 17α-Methyltestosterone underwent tissue restructuring and masculinization [44]. Alternatively, growth suppression occurs at maturity in males and may be an unintended consequence of masculinization in females [47,48,49]. Therefore, the masculinization process or other unobserved genetic and physiological changes in response to effluent exposure could affect the fish’s energy balance and somatic growth potential. 

Female mosquitofish collected from sites exposed to residential wastewater effluent displayed pronounced signs of masculinization, characterized by exhibiting elongation, fusion, and the alteration of anal fin rays 3, 4, and 5, which is typical of the male gonopodium [40]. The sperm-transmitting gonopodium constitutes a primary sexually dimorphic trait common in all live-bearing poeciliid fish [50], and changes are initiated by androgenic hormones produced by the testes [51,52]. The anal fin in typical females remains unmodified [53], but mature females display a recognizable gravid spot on the abdomen [54]. Both typical and masculinized females in our study displayed this spot, confirming their maturity. Our work was not able to determine whether masculinized fish can successfully reproduce or whether the feminization of males also occurs. Delayed cessation and the non-reversal of anal fin modifications when a small sample of fish was housed in clean water in the lab suggests that this permanent morphological change in females is due to chronic exposure. The persistent expression of male-like anatomical features in effluent-exposed female fish suggests that there may be other unobserved morphological or physiological effects on these fish. In roach (*Rutilus rutilus*), chronic exposure to treated wastewater effluent caused the feminization of males, as well as kidney, immune, and genetic changes, which were observable prior to the indicators of feminization [55]. 

Several instances of the reversal of sexually dimorphic morphology and physiology in fish in response to exogenous hormones and endocrine-disrupting chemicals have been documented [39,56]. In aquaculture, these effects are often targeted to maximize the production of desired stock [39]. In natural environments, the de-masculinization of male and the masculinization of female mosquitofish often occur in response to municipal sewage and industrial effluent [49,56,57,58,59], and this effect can be vary both spatially [51,58] and temporally [47]. Municipal sewage effluents in particular can contain multiple androgenic hormones [60] that have been shown to affect the expression of sexually dimorphic traits in mosquitofish, including triggering the development of gonopodium-like structures in females [40,41,44,48,51,52,61,62,63]. Additionally, lab studies have provided several insights into the causes and consequences of the androgenic stimulation of gonopodium formation in mosquitofish. For example, anal fin restructuring can begin in as little as 7–10 days of exposure, with mature structures visible by 50 days of exposure [44,61]. Although these changes can be triggered at any stage of development in live-bearing fish, including in embryos affected by maternal exposure [39], morphological changes may be more dramatic in younger fish [44]. Gonopodium-like structures do not lead to complete modification into the male morphology [40,48] and, similar to our observation, reversion may not happen following the removal of the stimulant [39,44]. Fish displaying reproductive reversal (either intersex gonad development or external gonopodium formation) have higher mortality and reduced fecundity [39,47,56], and in related Endler’s guppies (*Poecilia wingei*), intersex females are completely sterile [64]. Therefore, it is likely that the external changes in reproductive morphology associated with the OWTS-effluent-contaminated sites in St. Tammany and Iberia Parish are likely indicators of reduced mosquitofish long-term population viability and stability in these locations. 

To our knowledge, this is the first report of the extreme masculinization of female live-bearing fish not only in association with residential as opposed to industrial or municipal OWTS effluent, but also in the state of Louisiana. The minor restructuring of female mosquitofish anal fins was recently reported in northern Louisiana (Monroe, LA, USA), potentially in response to increased exposure to endocrine-disrupting chemicals near roadways and a golf course, but a consistent population-level effect could not be established [45]. Interestingly, a recent lab study utilizing fish obtained in south Louisiana (New Iberia, LA, USA) tested the effects of three known androgenic hormones, both individually and in mixed solutions, and did not find evidence of the development of intersex ovarian tissue [65], although it is unclear whether the fish were sourced from local natural reservoirs. Beyond mosquitofish, histological intersex conditions have been observed in largemouth bass (*Micropterus salmoides*) in central Louisiana (Alexandria, LA, USA) [66] and in three species of marine fish associated with hypoxic zones in the Gulf of Mexico off the coast of Louisiana [67], but only a small proportion of individuals examined displayed this trait and external morphological changes were not apparent due to minimal sexual dimorphism in these species. Additionally, although masculinization was suspected in longear sunfish (*Lepomis megalotis*) in the Pearl River (Bogalusa, LA, USA), the biochemical markers of endocrine activity did not support this hypothesis [68]. Therefore, intersex and masculinized fish appear to be rarely reported in the state, and our study is the first to note significant population-level masculinization effects in three species of native live-bearing fish in at least two Louisiana Parishes.

## 5. Conclusions

In summary, our study highlights the link between residential onsite wastewater treatment system (OWTS) effluent and reduced mosquitofish survivorship and predation effectiveness. While mosquitofish exhibit remarkable acute tolerance to effluent exposure, leading to potential biocontrol benefits in moderately contaminated areas, chronic exposure poses significant physiological complications, such as reduced prey capture behavior and long-term reproductive morphological changes indicative of endocrine disruption. The significant population-level masculinization effects observed in female fish from the effluent-exposed sites underscore the severity of this contamination. Our findings suggest that mosquitofish populations may thus struggle to persist in highly polluted areas and that their effectiveness as biocontrol agents may be compromised. Effective mosquito control through natural mosquitofish predation will therefore necessitate improved wastewater management practices. Continuing to address this phenomenon is crucial not only for the conservation of native fish populations, but also for understanding broader human and ecological repercussions in similar regions globally. 

## Figures and Tables

**Table 1:** Statistical results testing differences in water quality parameters across pairs of control and exposed sites in St. Tammany Parish. Pairs represent populations from which fish were collected on the same day.

	Pair 1: Mandeville (Control) and Slidell (Exposed)			Pair 2: Abita Springs (Control) and Covington (Exposed)		
Trait	r^2^	F_1,11_	*p*	r^2^	F_1,7_	*p*
pH	0.116	1.438	0.2557	0.040	0.289	0.6078
Oxidation-reduction potential (mV)	0.627	18.457	**0.0013**	0.003	0.024	0.8822
Conductivity (µS/cm)	0.802	44.646	**<0.0001**	0.092	0.709	0.4275
Salinity (psu)	0.818	49.294	**<0.0001**	0.059	0.440	0.5282
Total dissolved solids (mg/L)	0.805	45.417	**<0.0001**	0.057	0.422	0.5365
Temperature (°C)	0.029	0.328	0.5783	0.298	2.972	0.1284
Dissolved oxygen (mg/L)	0.055	0.645	0.4391	0.382	4.335	0.0758

Significant *p*-values following Benjamini–Hochberg correction of α = 0.05 are highlighted in bold.

**Table 2:** Statistical results testing differences in the female body condition parameters across pairs of control and exposed sites in St. Tammany Parish.

	Pair 1: Mandeville (Control) and Slidell (Exposed)			Pair 2: Abita Springs (Control) and Covington (Exposed)		
Trait	r^2^	F_1,47_	*p*	r^2^	F_1,48_	*p*
Standard Length (mm)	0.274	17.707	**0.0001**	0.054	2.746	0.104
Total Weight (g)	0.144	7.911	**0.0071**	0.013	0.636	0.429
Gonad Weight (g)	0.107	5.638	**0.0217**	0.036	1.793	0.1869
Somatic index (SI)	0.252	15.832	**0.0002**	0.090	4.723	0.0347
Somatic index, eviscerated (SI_E_)	0.211	12.554	**0.0009**	0.073	3.769	0.0581
Gonadosomatic index (GSI)	0.006	0.266	0.6085	0.002	0.084	0.7737

Significant *p*-values following Benjamini–Hochberg correction of α = 0.05 are highlighted in bold.

**Table 3:** Statistical results testing differences in the body condition parameters between masculinized and non-masculinized female fish in exposed sites.

Trait	r^2^	F_1,47_	*p*
Standard Length (mm)	0.155	8.629	**0.0051**
Total Weight (g)	0.130	7.008	**0.011**
Gonad Weight (g)	0.134	7.282	**0.0096**
Somatic index (SI)	0.001	0.052	0.8207
Somatic index, eviscerated (SI_E_)	0.004	0.211	0.648
Gonadosomatic index (GSI)	0.002	0.110	0.7417

Significant *p*-values following Benjamini–Hochberg correction of α = 0.05 are highlighted in bold.

**Table 4:** Statistical results testing differences in prey clearance across time points within each control population and concentration.

Population	Concentration (%)	Mean	SE	Min	Max	r^2^	F_4,35_	*p*
Mandeville (control)	0	98.3	1.66	86.7	100	0.92	94.32	**<0.0001**
	45	40.0	15.94	0	100	0.14	1.38	0.2593
	90	3.4	1.27	0	6.7	0.16	1.64	0.1865
Abita Springs (control)	0	97.5	2.50	80	100	0.84	46.19	**<0.0001**
	45	74.2	11.98	13.3	100	0.41	6.11	**0.0008**
	90	63.3	14.80	0	100	0.30	3.75	**0.0121**

SE is standard error. Significant *p*-values following Benjamini–Hochberg correction of α = 0.05 are highlighted in bold. Mean values are for prey clearance (%) at the final 60 min time point.

## Data Availability

Raw data are available as supplemental files alongside the manuscript.

## Supplementary Materials

The following supporting information can be downloaded at: https://www.mdpi.com/article/10.3390/toxics12040259/s1. Raw data.xlsx; Table S1: Summary of water quality parameters across populations; Table S2: Body condition metrics across populations; Table S3: Body condition metrics for fish used during toxicity assays; Figure S1: Overview of effluent toxicity assay tests; Figure S2: Temperature logger data during the toxicity assay experiments; Figure S3: Oxygen drawdown observed in each of 4 effluent concentrations, separated by test day and population tested; Table S4: Body condition metrics for fish used during prey capture tests.
