# Supplementary material for: Battle of the Bites: The Effect of Sewage Effluent Exposure on Mosquitofish Biocontrol of Mosquitoes in Residential Louisiana
Source: Toxics. 2024 Mar 30;12(4):259. doi: 10.3390/toxics12040259 (PMC11053664; doi:10.3390/toxics12040259)
Supplement: Supplementary file 1 [file toxics-12-00259-s001.zip › Supplement v1.pdf]

Field collection site water quality

Table S1 Summary of water quality parameters across populations.

		**Mandeville (control)** *n* = 5	**Abita Springs (control)** *n* = 4	**Slidell (exposed)** *n* = 10	**Covington (exposed)** *n* = 3	r^2	$F_{3,18}$	**p**
pH	Mean	8.01	8.10	7.48	8.33	0.14	1.02	0.4089
	SE	0.23	0.30	0.15	0.28			
	Min	6.92	7.10	7.20	7.77			
	Max	9.31	8.97	7.74	9.09			
Oxidation-reduction potential (mV)	Mean	45.34	134.60	-104.23	136.48	0.80	24.33	**<.0001**
	SE	12.84	9.03	51.52	7.62			
	Min	-60.63	122.35	-190.30	123.27			
	Max	74.05	169.60	-12.15	149.90			
Conductivity (µS/cm)	Mean	267.70	654.35	1064.75	475.10	0.60	9.11	**0.0007**
	SE	59.04	161.36	88.97	123.83			
	Min	5.60	263.55	920.00	176.47			
	Max	515.90	980.05	1226.75	697.95			
Salinity (psu)	Mean	0.123	0.286	0.500	0.225	0.63	10.24	**0.0004**
	SE	0.027	0.066	0.031	0.061			
	Min	0.000	0.120	0.460	0.080			
	Max	0.240	0.410	0.560	0.330			
Total dissolved solids (mg/L)	Mean	168.88	386.00	661.33	306.50	0.62	9.75	**0.0005**
	SE	37.42	88.01	39.85	80.55			
	Min	3.00	171.00	607.00	111.33			
	Max	331.00	556.00	739.00	448.00			
Temperature (°C)	Mean	26.61	28.87	27.30	25.57	0.23	1.76	0.1900
	SE	0.49	1.66	1.55	0.35			
	Min	24.44	25.08	24.22	24.92			
	Max	29.67	32.61	29.17	26.52			
Dissolved oxygen (mg/L)	Mean	5.51	8.17	4.19	4.40	0.27	2.23	0.1198
	SE	0.68	1.14	2.14	1.44			
	Min	2.78	4.29	1.55	1.86			
	Max	9.63	10.69	8.43	8.47			

SE is standard error. Significant p-values following Benjamini-Hochberg correction of $\alpha = 0.05$ are highlighted in bold. Statistical results show tests of differences across all 4 populations.

Body condition

Table S2 Body condition metrics across populations.

		**Mandeville (control)** *n* = 25	**Abita Springs (control)** *n* = 25	**Slidell (exposed)** *n* = 24	**Covington (exposed)** *n* = 25	r^2	$F_{3,95}$	**p**
Standard Length (mm)	Mean	29.05	27.51	24.59	25.55	0.17	6.26	**0.0006**
	SE	0.801	0.711	0.687	0.947			
	Min	23.16	22.13	20.43	18.60			
	Max	40.85	40.22	33.95	35.26			
Total Weight (g)	Mean	0.391	0.310	0.262	0.267	0.08	2.84	0.0418
	SE	0.0367	0.0452	0.0269	0.0294			
	Min	0.177	0.152	0.135	0.120			
	Max	1.016	1.307	0.749	0.643			
Gonad Weight (g)	Mean	0.066	0.046	0.044	0.036	0.13	4.91	**0.0032**
	SE	0.0077	0.0061	0.0047	0.0037			
	Min	0.019	0.021	0.021	0.011			
	Max	0.168	0.170	0.101	0.081			
Somatic index (SI)	Mean	0.0015	0.0014	0.0017	0.0015	0.25	10.64	**<0.0001**
	SE	0.000027	0.000047	0.000028	0.000044			
	Min	0.0011	0.0009	0.0014	0.0010			
	Max	0.0017	0.0020	0.0019	0.0019			
Somatic index, eviscerated (SI_E)	Mean	0.0012	0.0012	0.0014	0.0013	0.17	6.38	**0.0005**
	SE	0.000019	0.000047	0.000028	0.000039			
	Min	0.0010	0.0007	0.0011	0.0009			
	Max	0.0014	0.0017	0.0017	0.0017			
Gonado-somatic index (GSI)	Mean	16.69	15.39	17.53	14.90	0.03	1.04	0.3765
	SE	1.121	1.018	1.158	1.350			
	Min	5.03	8.48	9.81	5.41			
	Max	27.02	29.34	30.82	31.08			

SE is standard error. Significant p-values following Benjamini-Hochberg correction of $\alpha = 0.05$ are highlighted in bold. Statistical results show tests of differences across all 4 populations.

Toxicity assays

Table S3 Body condition metrics for fish used during toxicity assays.

		**Mandeville (control)** *n* = 104	**Abita Springs (control)** *n* = 102	**r^2**	**t_{204}**	**p**
Standard length (mm)	Mean	30.08	30.60	0.002709	-0.74446	0.4575
	SE	0.491	0.505			
	Min	20.96	18.33			
	Max	42.70	43.34			
Weight (g)	Mean	0.552	0.557	0.000069	-0.1189	0.9055
	SE	0.0272	0.0311			
	Min	0.148	0.111			
	Max	1.568	1.596			
Somatic index	Mean	0.0019	0.0018	0.048933	3.239757	**0.0014**
	SE	0.000034	0.000023			
	Min	0.0010	0.0012			
	Max	0.0032	0.0023			
SE is standard error. Significant p-values following Benjamini-Hochberg correction of α = 0.05 are highlighted in bold. Statistical results show tests of differences across control populations.						

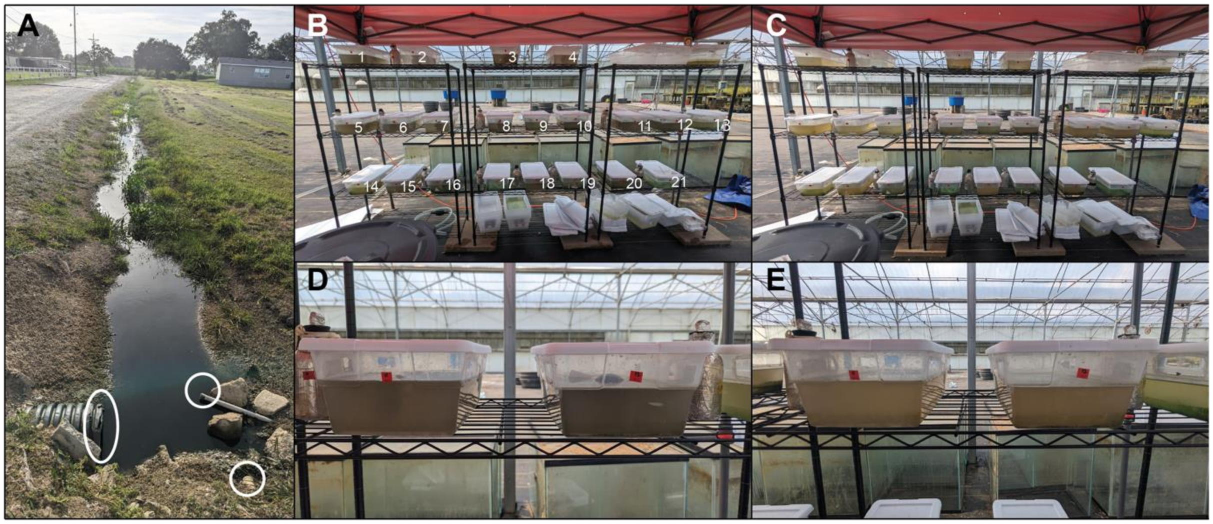

Figure S1 Overview of effluent toxicity assay tests. A) The source drainage ditch where effluent water was collected for the toxicity and feeding assays. White circles indicate the septic effluent input sources. B-C) Overview of test bins set up at the Ecology center at time 0 (B) and 24 hours (C). Numbers show the ordering scheme of bins. In this test, bins contained the following concentrations: 1: 60%; 2: 0%; 3: 60%; 4: 30%; 5: 75%; 6: 45%; 7: 30%; 8: 45%; 9: 30%; 10: 90%; 11: 75%; 12: 90%; 13: 15%; 14: 15%; 15: 60%; 16: 45%; 17: 0%; 18: 75%; 19: 15%; 20: 90%; 21: 0%. D-E) Closeup of tanks 11 and 12 at time 0 (D) and 24 hours (E) showing the color change after 24 hours.

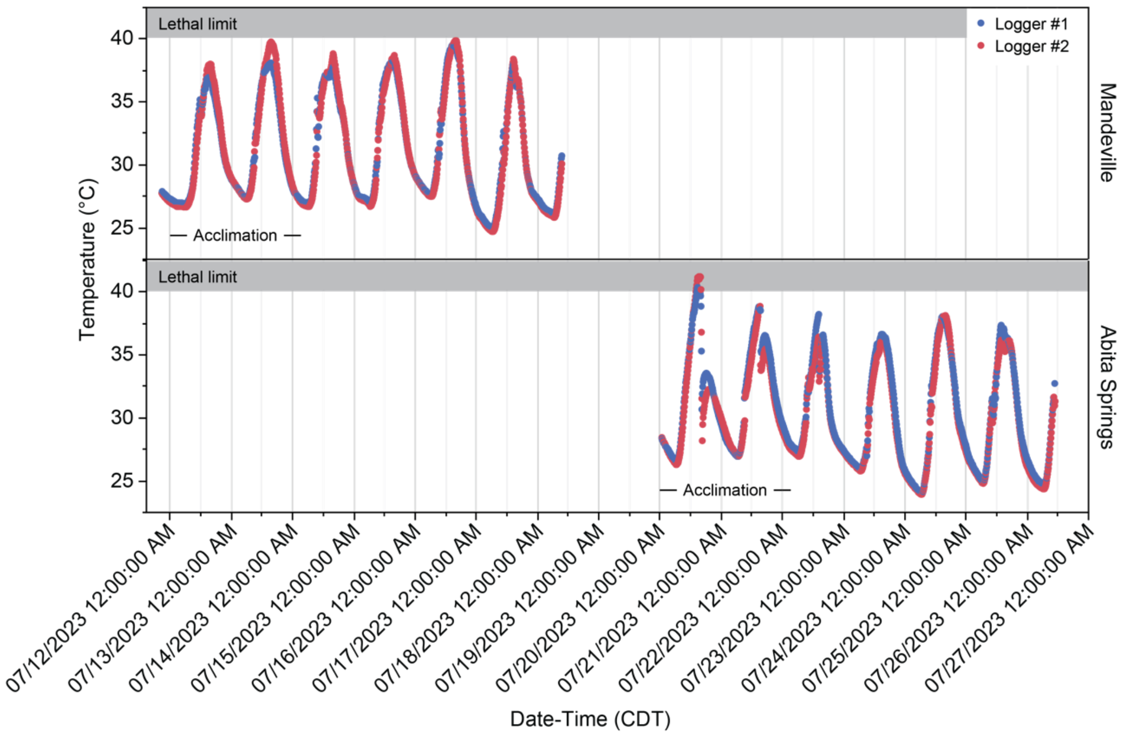

Figure S2 Temperature logger data during the toxicity assay experiments. Two temperature loggers were placed on opposite ends of the shelving, inside control (0% effluent) test bins. Daily temperatures inside test bins were similar between loggers. Extreme high temperatures approaching the lethal maximum were observed during the second test with Abita Springs fish and ice was added at midday to lower temperatures for each day of acclimation as well as the first test day.

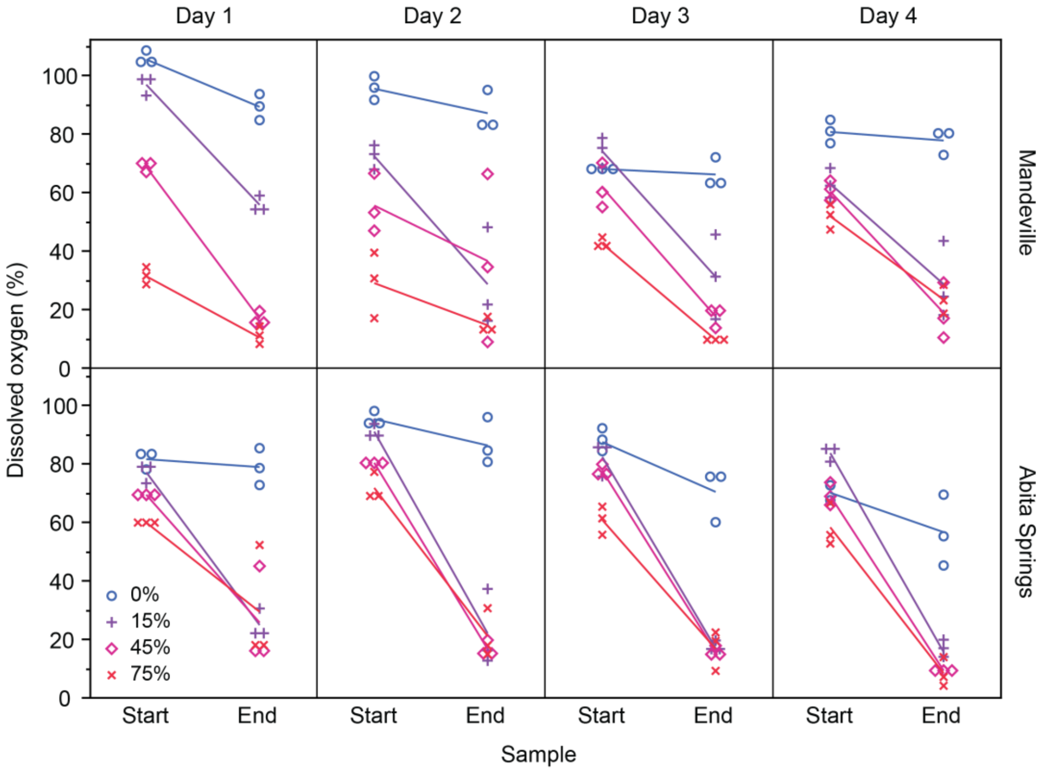

Figure S3 Oxygen drawdown observed in each of 4 effluent concentrations, separated by test day and population tested. Colors and shapes correspond to concentrations. Lines connect the mean values for each concentration. Starting oxygen levels were lowest in the highest effluent concentration and drawdown was highest at intermediate concentrations. These patterns were consistent across all 8 days measured.

Prey capture trials

Table S4 Body condition metrics for fish used during prey capture tests.

Population	Day	Concentration (%)	# fish	Mean	SE	Min	Max
Mandeville	1	0	4	31.63	2.871	24.51	37.82
	1	45	4	26.63	2.305	19.77	29.72
	1	90	4	26.96	1.971	23.51	32.36
	Day 1 combined		12	28.41	1.435	19.77	37.82
	2	0	4	33.22	2.621	26.37	39.1
	2	45	4	33.42	1.487	29.6	36.17
	2	90	4	25.07	0.344	24.38	25.73
	Day 2 combined		12	30.57	1.487	24.38	39.1
	Mandeville combined		24	29.49	1.035	19.77	39.1
Abita Springs	1	0	4	34.87	0.726	33.56	36.91
	1	45	4	33.04	1.938	29.49	38.56
	1	90	4	27.25	1.159	25.25	30.48
	Day 1 combined		12	31.72	1.212	25.25	38.56
	2	0	4	31.49	1.324	28.71	34.24
	2	45	4	30.61	3.573	22.47	39.65
	2	90	4	29.85	1.543	26.86	33.97
	Day 2 combined		12	30.65	1.256	22.47	39.65
	Abita Springs combined		24	31.18	0.861	22.47	39.65
SE is standard error.							
